# Circulating Tumour Cell *GALNT13* Expression Is Associated with Prognosis and Treatment Response in Non-Small Cell Lung Cancer

**DOI:** 10.3390/ijms27209095

**Published:** 2026-10-14

**Authors:** Eugenia Fernández Armúa, María José Serrano, Diego Santana, Mariana Carrasco, María Pilar Molina, Inés Aznar, Marcos Acosta, Beatriz Villar, Juan Ferrari, Diego Touya, Eduardo Osinaga, Nora Berois

**Affiliations:** 1 Laboratorio de Oncología Molecular, Servicio de Oncología, Hospital Maciel, Administración de Servicios de Salud del Estado (ASSE), 25 de Mayo 174, Montevideo 11000, Uruguay; eugenia.ferarm@gmail.com (E.F.A.); dsantana.8@outlook.com (D.S.); marianacarrascoalosilla@gmail.com (M.C.); fedeacostav@gmail.com (M.A.); dtouya@gmail.com (D.T.); eosinaga@pasteur.edu.uy (E.O.); 2 Laboratorio de Glicobiología e Inmunología Tumoral, Institut Pasteur de Montevideo, Mataojo 2020, Montevideo 11400, Uruguay; 3 Liquid Biopsy and Cancer Interception Group, Centre for Genomics and Oncological Research (GENyO), Pfizer/University of Granada/Andalusian Regional Government, PTS Granada, Avenida de la Ilustración 114, 18016 Granada, Spain; mjose.serrano@genyo.es (M.J.S.); p.molinavallejo@gmail.com (M.P.M.); ines.aznar@genyo.es (I.A.); 4 Pathological Anatomy Unit (UPIGAP), University Hospital Virgen de las Nieves, Av. de las Fuerzas Armadas 2, 18014 Granada, Spain; 5 Instituto de Investigación Biosanitaria ibs.GRANADA, Av. de Madrid 15, Beiro, 18012 Granada, Spain; 6 Servicio de Hemoterapia, Hospital Maciel, ASSE, 25 de Mayo 174, Montevideo 11000, Uruguay; 7 Departamento de Inmunobiología, Facultad de Medicina, Universidad de la República, Av. General Flores 2125, Montevideo 11800, Uruguay

**Keywords:** cancer, O-glycosylation, non-small cell lung cancer (NSCLC), liquid biopsy, *GALNT13*, biomarker, circulating tumour cells (CTCs)

## Abstract

Reliable circulating biomarkers for prognosis and treatment monitoring in non-small cell lung cancer (NSCLC) remain limited. Liquid biopsy offers a minimally invasive approach to identify clinically informative tumour-derived markers. Given the emerging role of GalNAc-transferases in cancer biology, we evaluated *GALNT13* expression in peripheral blood as a potential biomarker for prognosis and disease monitoring in lung cancer patients. Sensitive molecular assays (nested and digital RT-PCR) were developed to detect *GALNT13* mRNA in circulating tumour cells (CTCs). These methods were applied to peripheral blood samples from non-small cell lung cancer patients to assess prognostic value and enable longitudinal monitoring. A cohort of 143 NSCLC patients was analysed, of whom 60% were *GALNT13*-positive in peripheral blood at baseline. During a five-year follow-up, *GALNT13* positivity was significantly associated with reduced overall survival. In several patients, increases in *GALNT13* levels preceded radiologic evidence of disease progression. *GALNT13* expression in CTCs emerges as a promising biomarker for prognosis and treatment monitoring in NSCLC. Prospective studies in larger cohorts are warranted to validate its clinical utility.

## 1. Introduction

Lung cancer remains one of the most frequently diagnosed malignancies worldwide and the leading cause of cancer-related death [1]. Its management remains challenging due to late diagnosis, significant molecular heterogeneity, and rapid disease progression. Despite advances in targeted therapies and immunotherapy, overall survival—particularly in advanced disease—remains poor [2]. The lack of reliable tools for early detection and real-time treatment monitoring continues to limit timely clinical intervention [3]. Therefore, accurate, minimally invasive, and clinically actionable biomarkers are urgently needed to improve diagnosis, patient stratification, and personalised treatment strategies.

Liquid biopsy has emerged as a powerful tool in precision oncology, enabling the detection of tumour-derived components in body fluids and fostering novel diagnostic approaches [4]. Its minimal invasiveness, accessibility, and suitability for serial sampling make it particularly valuable when tissue biopsy is unavailable or insufficient. Several circulating analytes have been explored as potential biomarkers, including circulating tumour cells (CTCs), circulating tumour DNA (ctDNA), RNA, extracellular vesicles (EVs), and tumour-educated platelets [5]. Among these, CTCs are thought to play a central role in the metastatic cascade and provide a dynamic window into tumour heterogeneity and evolution. Over the past two decades, extensive research has led to the approval of CTCs enumeration using the CellSearch^®^ system as a prognostic tool in breast, prostate and colorectal cancers [6]. However, tumours are dynamic and heterogeneous entities, and molecular characterisation of CTCs provides insights that extend beyond simple enumeration [7].

Aberrant glycosylation is increasingly recognised as a hallmark of cancer progression [8]. Incomplete *O*-glycosylation exposes tumour-associated carbohydrate antigens that influence cell–cell interactions and tumour development. The Tn antigen (GalNAcα-*O*-Ser/Thr) is the simplest *O*-glycan, consisting of a single N-acetylgalactosamine residue linked to serine or threonine residues. Its synthesis is catalysed by the GalNAc-transferase (GalNAc-T) family (EC 2.4.1.41), which comprises 20 isoenzymes [9], several of which have been implicated in tumour progression and proposed as cancer biomarkers [10,11,12,13,14]. Our group identified GalNAc-T6 as a biomarker in breast [15] and colon cancer [16] and demonstrated prognostic roles for *GALNT13* and *GALNT9* in neuroblastoma [17,18] and for GalNAc-T13 in breast cancer [19]. Growing evidence indicates that GalNAc-T family members, particularly GalNAc-T13, contribute to lung cancer progression, promoting metastasis and correlating with poor prognosis [20]. Considering the expanding role of liquid biopsy in monitoring lung cancer and the lack of suitable biomarkers, we investigated *GALNT13* mRNA as a potential novel biomarker in non-small cell lung cancer (NSCLC) patients. In this work, we aimed to assess the expression of this gene in CTCs through in vitro experiments using cell lines and validation in peripheral blood samples from lung cancer patients. The results obtained during patient follow-up support its potential utility for monitoring treatment throughout the course of the disease.

## 2. Results

### 2.1. GALNT13 Expression in Lung Cancer Cell Lines

**Figure 1:**
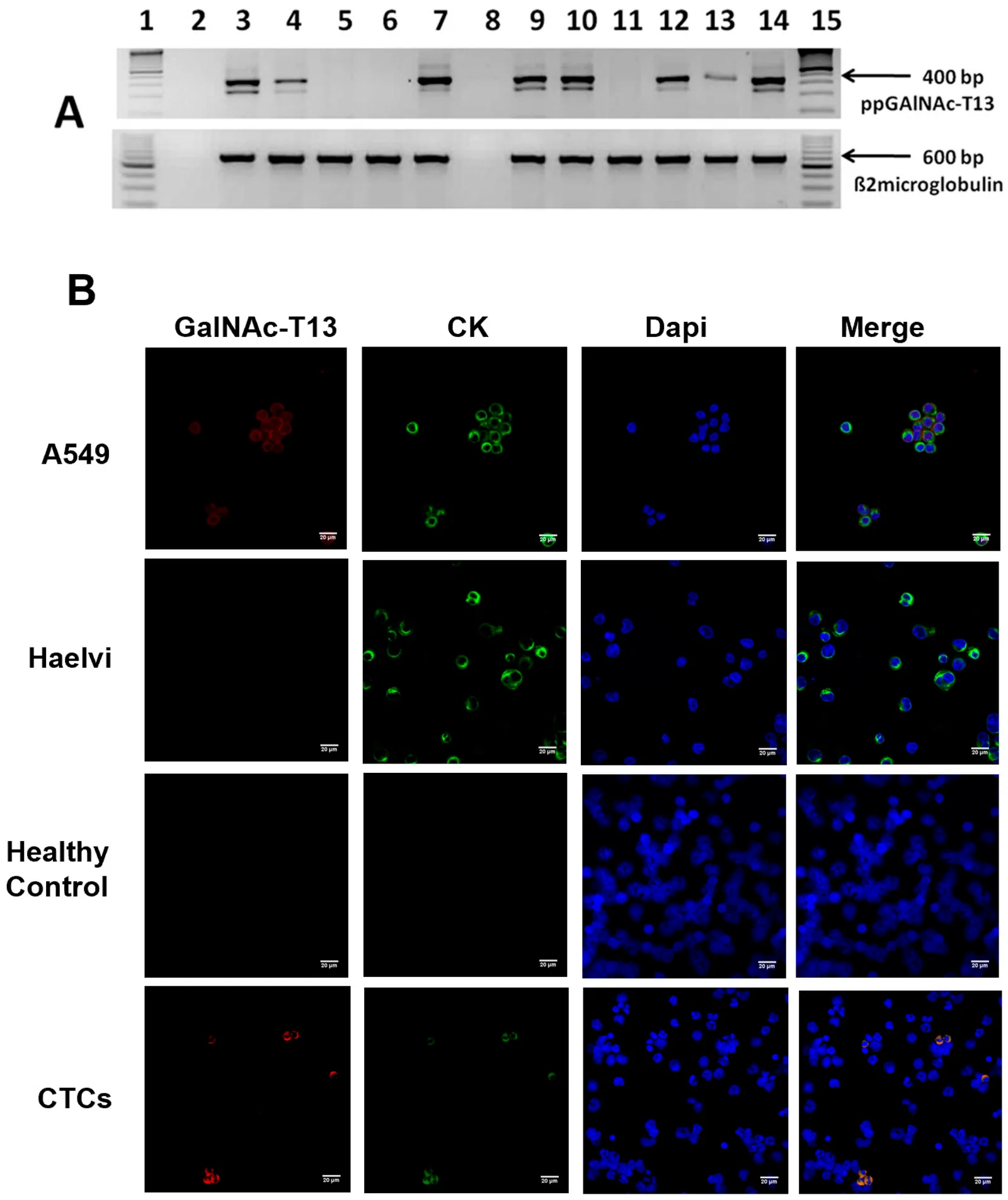
*GALNT13* expression in lung cancer cell lines and circulating tumour cells. (**A**) RT-PCR analysis of *GALNT13* mRNA expression in lung cancer cell lines. Lane 1, 100-bp DNA ladder; lane 2, negative control (NTC); lanes 3–7, lung cancer cell lines (NCI-H1703, NCI-H526, NCI-H838, SK-MES1, H69AR); lane 8, negative control (H2O); lanes 9–12, lung cancer cell lines (NCI-H1755, A549, NCI-H1975, NCI-H1650); lane 13, NL-20 bronchial epithelial cells; lane 14, positive control (BM cell line); lane 15, 100-bp DNA ladder. (**B**) Indirect immunofluorescence analysis of A549 cells, immortalised human alveolar epithelial cells (hAELVi), peripheral blood cells from healthy donors, and circulating tumour cells (CTCs) from non-small cell lung cancer (NSCLC) patients. GalNAc-T13 is shown in red (Alexa Fluor 555), cytokeratin in green (FITC), and nuclei in blue (Hoechst 33342). Merged images show co-localisation of cytokeratin and GalNAc-T13. (Scale bar = 20 μm; magnification = 63×).

We previously reported that a nested RT-PCR assay enabled detection of *GALNT13* mRNA in bone marrow from metastatic neuroblastoma patients but not in healthy controls [17]. More recently, we applied the same technique to demonstrate *GALNT13* expression in breast cancer cell lines [19]. In the present work, prior to analysing clinical samples, we assessed *GALNT13* expression in a panel of human lung cancer cell lines to determine whether the enzyme is expressed across different lung cancer subtypes. We found *GALNT13* mRNA in three adenocarcinoma (ADC) cell lines (A549, NCI-H1755, and NCI-H1650), both small cell lung cancer (SCLC) cell lines (NCI-H526 and H69AR), and one squamous cell carcinoma (SCC) cell line (NCI-H1703), with weaker expression also detected in the immortalised non-tumorigenic bronchial epithelial cell line NL-20 (Table 1; Figure 1A). In contrast, RT-PCR was negative in the SCC cell line SK-MES-1 and in two ADC lines (NCI-H838 and NCI-H1975). Multiple bands were observed by RT-PCR (Figure 1A), suggesting the presence of *GALNT13* splice variants in lung cancer, consistent with previous identification of multiple isoforms of this enzyme in neuroblastoma [21].

### 2.2. Single-Cell Validation of GALNT13 Expression in CTCs Through IsoFlux™ Isolation and Confocal Analysis

To determine whether *GALNT13* expression detected by PCR originated specifically from CTCs, we performed CTC enrichment using IsoFlux™ followed by immunostaining and confocal microscopy. To validate the immunofluorescence assay, the A549 lung cancer cell line (*GALNT13*-positive) and hAELVi (*GALNT13*-negative) cells were used as controls. Co-expression of GalNAc-T13 and cytokeratin was assessed, with cytokeratin serving as a marker of epithelial origin to distinguish cancer cells from peripheral blood mononuclear cells. As expected, A549 cells expressed both *GALNT13* and cytokeratin, whereas hAELVi cells expressed cytokeratin only (Figure 1B).

IsoFlux™-enriched CTCs from 11 patients with advanced NSCLC were then examined. All patients had cytokeratin-positive CTCs, while 9/11 were positive for GalNAc-T13. These results indicate that *GALNT13* protein expression is restricted to a subset of circulating tumour cells, in agreement with the RT-PCR results showing that not all lung cancer cell lines expressed the enzyme. Peripheral blood mononuclear cells from four healthy donors were also analysed; none showed GalNAc-T13 or cytokeratin staining, confirming the absence of detectable background expression in non-tumour samples.

### 2.3. Optimisation of GALNT13 mRNA Detection

The analytical performance of nRT-PCR and quantitative dRT-PCR for detecting *GALNT13* mRNA was evaluated using a dilution model simulating rare CTCs in peripheral blood. *GALNT13*-expressing A549 cells were mixed with *GALNT13*-negative K562 cells at ratios ranging from 1:100 to 1:204,800. As shown in Table 2, the detection limits obtained were 0.01% for nRT-PCR and 0.02% for dRT-PCR. Specificity of the nRT-PCR assay was confirmed using peripheral blood samples from 48 healthy donors, all of which tested negative for *GALNT13*.

### 2.4. Baseline CTC-GALNT13+ and Overall Survival

**Figure 2:**
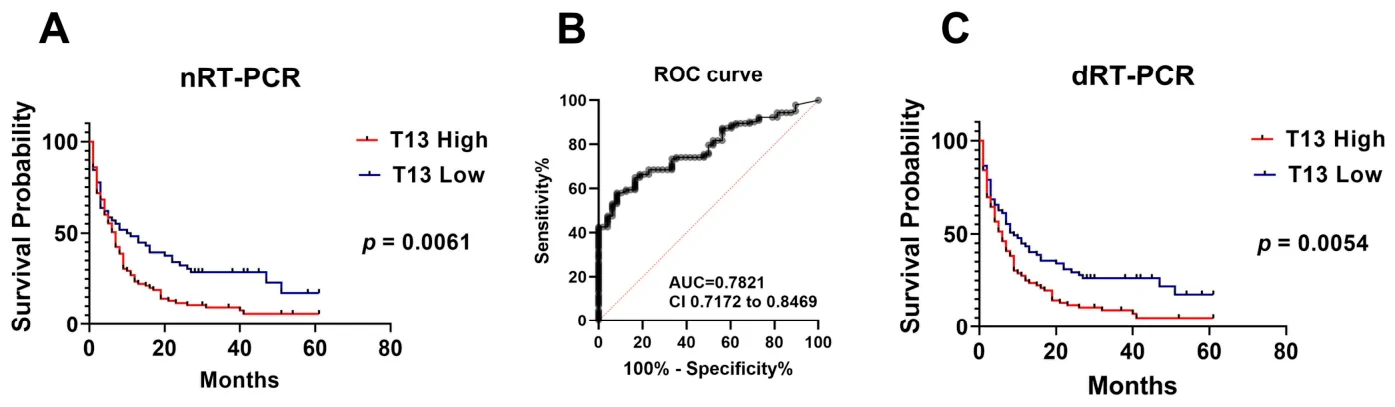
Prognostic value of *GALNT13* expression in peripheral blood from NSCLC patients. (**A**) Kaplan–Meier overall survival curves according to *GALNT13* expression detected by nested RT-PCR (log-rank test, *p* = 0.0061). (**B**) Receiver operating characteristic (ROC) curve for *GALNT13* detection by digital RT-PCR (AUC, 0.7811; 95% CI, 0.7161–0.8461). (**C**) Kaplan–Meier overall survival curves according to *GALNT13* expression measured by digital RT-PCR (log-rank test, *p* = 0.0054).

*GALNT13* expression was analysed by nRT-PCR in peripheral blood from 143 NSCLC patients (mean age, 65 years; range 32–84; 94 men and 49 women). The follow-up period—lasting 5 years or until the patient’s death (126 cases)—had a median duration of 7 months. CTC-*GALNT13*+ cells were detected in 86 of 143 (60%) baseline samples. By histology, positivity was observed in 45 of 76 (59%) ADC, 26 of 39 (67%) SCC and 15 of 28 (54%) other histologies, with no significant difference among groups. By stage, positivity was detected in 18 of 36 (50%) patients with stage I–IIIA and 68 of 107 (64%) advanced cases, stage IIIB–IV (Table 3). *GALNT13* expression was significantly correlated with tumour size (*p* = 0.027) but not with lymph node or metastasis status. During a 5-year follow-up, *GALNT13* expression detected by nRT-PCR was significantly associated with reduced overall survival (log-rank test, *p* = 0.0061; Figure 2A). In a multivariable Cox regression model adjusted for disease stage, age, sex, and smoking status, *GALNT13* positivity remained significantly associated with shorter overall survival (HR 1.56, 95% CI 1.05–2.31; *p* = 0.028).

Receiver operating characteristic (ROC) curve analysis was performed to assess the diagnostic performance of *GALNT13* detection by dRT-PCR using the full NSCLC cohort (n = 143) and samples from 48 healthy controls from the Department of Hemotherapy. Based on the ROC analysis, the cutoff of 0.5 copies/uL was selected for dRT-PCR based on the sensitivity and specificity obtained. The biomarker showed an AUC value of 0.7811 (95% CI: 0.7161–0.8461) (Figure 2B), with a sensitivity of 53.15% and a specificity of 93%.

The distribution of CTC-*GALNT13*+ detected by dRT-PCR showed similar correlations with clinicopathologic variables as those obtained by nRT-PCR regarding sex, histology and TNM (tumour size, *p* = 0.045). In contrast, positivity by dRT-PCR was significantly higher in advanced diseases compared with early-stage cases (59% vs. 36%, respectively, *p* = 0.011) (Table 3). Kaplan-Meier also demonstrated significantly lower overall survival in *GALNT13*-positive patients (*p* = 0.0054; Figure 2C). Consistently, in the corresponding multivariable Cox regression model adjusted for clinically relevant factors, *GALNT13* positivity detected by dRT-PCR also remained significantly associated with shorter overall survival (aHR 1.49, 95% CI 1.02–2.18; *p* = 0.039).

### 2.5. GALNT13 mRNA for Treatment Monitoring

**Figure 3:**
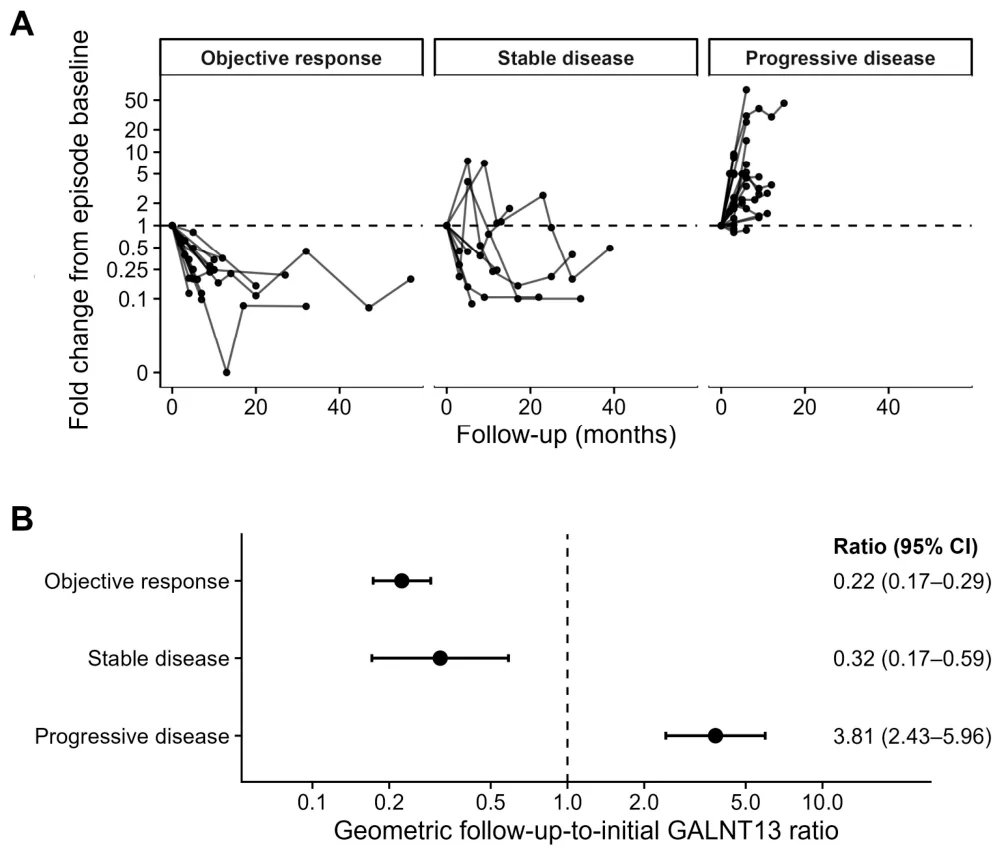
**Longitudinal changes in *GALNT13* expression by clinical disease status.** (**A**) Longitudinal trajectories of *GALNT13* fold change during evaluable clinical episodes, shown separately for objective response, stable disease, and progressive disease. Each point represents a blood sample, and each line represents an evaluable clinical episode. Individual patients could contribute more than one episode and therefore appear in multiple clinical-status groups. Values were normalised to the first sample of each episode (fold change = 1); a pseudo-logarithmic *y*-axis was used for visualisation. (**B**) Generalised estimating equation (GEE) analysis accounting for within-patient correlation across 49 evaluable paired comparisons from 34 patients. Results are expressed as geometric follow-up-to-initial ratios (95% CI): objective response, 0.22 (0.17–0.29; *p* < 0.001); stable disease, 0.32 (0.17–0.59; *p* < 0.001); and progressive disease, 3.81 (2.43–5.96; *p* < 0.001). The vertical dashed line indicates a ratio of 1.

Longitudinal monitoring of 45 NSCLC patients (1–9 samples per patient) showed that *GALNT13* expression measured by dRT-PCR varied according to clinical status during follow-up. Clinical status was classified as stable disease (S), objective response (R), or progressive disease (P), based on imaging findings from PET-CT (Positron Emission Tomography–Computed Tomography), computed tomography, and/or bone scintigraphy. *GALNT13* expression was quantified as fold change relative to baseline and as copy numbers per μL. Eleven patients remained *GALNT13*-negative throughout follow-up and were therefore excluded from quantitative paired comparisons. The remaining 34 patients contributed 49 pairs to the repeated-measures analysis. Because some patients contributed more than one pair, longitudinal changes in *GALNT13* expression were analysed using generalised estimating equations (GEE) with patient-level clustering. *GALNT13* expression decreased significantly during objective response, with a geometric follow-up-to-initial ratio of 0.22 (95% CI 0.17–0.29; *p* < 0.001). A significant decrease was also observed during stable disease (ratio 0.32, 95% CI 0.17–0.59; *p* < 0.001). In contrast, disease progression was associated with a marked increase in *GALNT13* expression (ratio 3.81, 95% CI 2.43–5.96; *p* < 0.001) (Figure 3).

**Figure 4:**
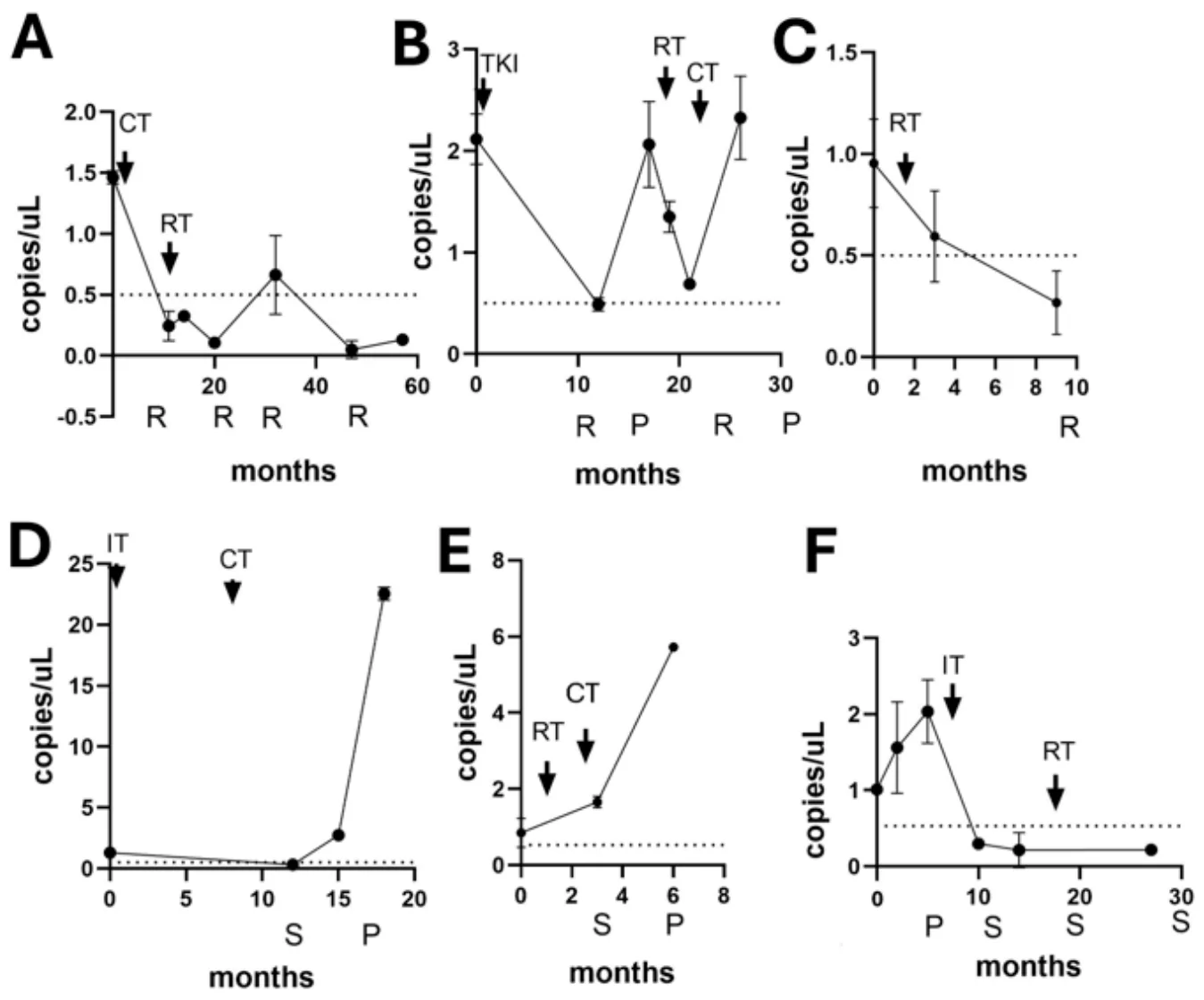
Longitudinal monitoring of *GALNT13* expression in representative NSCLC patients receiving systemic therapy. *GALNT13* levels measured by digital RT-PCR are shown: (**A**) LOM12; (**B**) LOM17; (**C**) LOM46; (**D**) LOM50; (**E**) LOM54; (**F**) LOM89. Treatment modalities are indicated as CT (chemotherapy), RT (radiotherapy), TKI (tyrosine kinase inhibitor), and IT (immunotherapy). Clinical status was defined according to imaging evaluation as R (objective response), S (stable disease), or P (progressive disease).

Six representative cases are shown as examples of treatment monitoring (Figure 4). All were positive at baseline by both methods. Patient LOM12 (Figure 4A), with stage IV ADC, exhibited response to treatment following chemotherapy and radiotherapy, with *GALNT13* expression falling below the cutoff value. Patient LOM17 (Figure 4B), also with stage IV ADC harbouring an EGFR exon19 deletion, was treated with tyrosine kinase inhibitors, which coincided with a decrease in *GALNT13* levels. One year later, *GALNT13* expression rose again, preceding clinical progression, but declined following radiotherapy and chemotherapy. A final increase was observed prior to disease progression, and the patient died. Patient LOM46 (Figure 4C), with stage IIIA SCC, received radiotherapy and showed both disease objective response and a decrease in *GALNT13* expression. Patients LOM50 (Figure 4D) and LOM54 (Figure 4E), both with stage IV disease, showed periods of stable disease during treatment, with *GALNT13* levels remaining near baseline. However, a clear increase preceded disease progression in both cases. Finally, patient LOM89 (Figure 4F) was recruited during disease progression and exhibited elevated *GALNT13* expression. Owing to PD-L1 tumour expression, the patient received immunotherapy followed by radiotherapy, achieving stable disease and a biomarker decrease below the cutoff level. These cases illustrate the potential utility of *GALNT13* expression as a biomarker for disease monitoring in lung cancer.

## 3. Discussion

Liquid biopsy has emerged as a key component of precision oncology, providing minimally invasive access to tumour-derived biomarkers that may improve cancer diagnosis, prognostic assessment and treatment monitoring. In NSCLC, CTCs represent an attractive source of tumour material, offering complementary information to tissue-based approaches [22]. Although the CellSearch^®^ system—the only FDA-cleared platform for CTC enumeration—has shown prognostic value in several tumour types, the low abundance of CTCs and variable EpCAM expression associated with epithelial–mesenchymal transition diminishes its performance [23]. Despite more than three decades of research, no CTC-based assay has yet been widely adopted in clinical practice for lung cancer, highlighting the need for robust and clinically informative biomarkers.

Molecular detection of tissue- or tumour-specific transcripts in whole blood has therefore emerged as an alternative strategy, and several biomarkers—including CEA, CK19, c-met, EpCAM and MAGE-3—have been evaluated [24]. However, contradictory results have been reported, highlighting technical pitfalls, whereby high specificity can often be achieved only at the expense of reduced sensitivity, representing a major limitation when attempting to identify rare events [25]. Although multiple ongoing clinical trials continue to assess these approaches, issues related to standardisation still hinder their incorporation into routine clinical practice.

Aberrant glycosylation is increasingly recognised as a hallmark of cancer progression, influencing cell signalling, immune evasion, and metastatic dissemination. Members of the GalNAc-transferase family initiate mucin-type *O*-glycosylation and have been implicated in tumour development across multiple cancer types. Among them, *GALNT13* was described as a neural-specific enzyme, involved in neuronal differentiation [26,27,28], but subsequent studies have reported aberrant expression in several malignancies, including neuroblastoma [17], lung cancer [29], and breast cancer [19], where it has been associated with tumour behaviour.

Our results extend these previous observations by demonstrating that *GALNT13* expression can also be detected in CTCs from NSCLC patients and may serve as a clinically informative biomarker in the context of liquid biopsy. Using highly sensitive and specific molecular approaches, we demonstrated that *GALNT13* expression can be detected in peripheral blood and is significantly associated with overall survival. Notably, this association remained statistically significant after adjustment for clinically relevant factors, with consistent findings for *GALNT13* positivity determined by both nRT-PCR and dRT-PCR. These results are consistent with prior evidence linking GalNAc-T13 to metastatic potential. In murine lung models, Matsumoto et al. demonstrated more aggressive behaviour through the expression of trimeric Tn antigen (GalNAc-*O*-Ser/Thr) on syndecan [30,31]. These findings are further supported by evidence showing that Tn expression in a similar model promotes immunosuppression and angiogenesis, thereby favouring tumour progression [32].

Moreover, longitudinal monitoring revealed a clear association between changes in *GALNT13* expression and clinical disease status. Expression decreased during objective response and stable disease, whereas disease progression was associated with an approximately 3.8-fold increase relative to the initial sample in each pair. These findings support the possibility that longitudinal *GALNT13* measurements may reflect changes in tumour burden and treatment response. Importantly, in several individual patients, increases in *GALNT13* levels preceded radiologic evidence of disease progression. Such patterns support the potential of *GALNT13* as a dynamic biomarker for disease monitoring, although prospective validation using predefined sampling intervals and standardised response assessments will be required before its clinical utility can be established.

Although our study complies with REMARK guidelines [33], like many translational biomarker studies, our work has several limitations. First, the patient cohort was derived primarily from a single clinical centre, and independent validation in larger multicenter cohorts will be required. Second, although *GALNT13* expression was confirmed at the protein level in CTCs, the number of samples analysed using the IsoFlux enrichment platform was limited. Finally, while our findings support an association between *GALNT13* expression and tumour aggressiveness, the mechanistic role of this glycosyltransferase in NSCLC progression remains to be fully elucidated.

In conclusion, our preliminary and exploratory evidence supports the potential diagnostic, prognostic, and treatment-monitoring relevance of *GALNT13* expression in CTCs as a promising biomarker in NSCLC. These findings should be interpreted in the context of the growing clinical interest in liquid biopsy–based biomarkers for lung cancer. The combination of highly sensitive nested PCR for detection and digital PCR for quantitative monitoring provides a practical strategy that could be implemented using widely available molecular technologies. Integration of *GALNT13* analysis with other liquid biopsy approaches, such as circulating tumour DNA profiling, may further improve patient stratification and treatment monitoring. Future prospective multicentre studies will be essential to validate these findings and to define the clinical utility of *GALNT13* within the evolving landscape of precision oncology.

## 4. Materials and Methods

### 4.1. Patients and Clinical Samples

Non-small cell lung cancer (NSCLC) patients treated at the Oncology Service, Maciel Hospital (ASSE, Montevideo, Uruguay) (n = 143) were prospectively enrolled at first consultation for the evaluation of *GALNT13* expression in peripheral blood between August 2020 and August 2024. In parallel, peripheral blood samples from 48 healthy donors (18–76 years old; 25 female, 23 male) were collected as controls at the Hemotherapy Department, Maciel Hospital. Additionally, peripheral blood samples from 11 NSCLC patients and 4 healthy donors collected at the Hospital Universitario Virgen de las Nieves (HUVN), Granada, Spain, were included specifically for Circulating Tumour Cell (CTC) enrichment using the IsoFlux™ system (Fluxion Biosciences, Alameda, CA, USA).

Peripheral blood samples collected in EDTA tubes were processed within 4 h. The buffy coat fraction was isolated by centrifugation at 350× *g*. Residual red blood cells were lysed using EL buffer (Qiagen, Hilden, Germany) and white blood cells were washed with PBS. The resulting pellet (washed buffy coat) was fractionated for molecular biology assays, resuspended in Trizol^TM^ Reagent (Thermo Fisher Scientific, Waltham, MA, USA), and stored at −80 °C until further use, or fixed in 4% paraformaldehyde and stored in PBS containing 1% BSA, 10 mM EDTA, and 0.01% sodium azide for immunophenotyping.

### 4.2. Cell Lines

The human lung cancer cell lines used in this study—A549 (RRID:CVCL_0023), SK-MES-1 (RRID:CVCL_0630), NCI-H1703 (RRID:CVCL_1490), NCI-H838 (RRID:CVCL_1594), NCI-H1755 (RRID:CVCL_1492), NCI-H526 (RRID:CVCL_1569), NCI-H1650 (RRID:CVCL_1483), NCI-H1975 (RRID:CVCL_1511), H69AR (RRID:CVCL_3513) and immortalized human bronchial epithelial cell line NL-20 (RRID:CVCL_3756)—as well as the chronic myelogenous leukaemia cell line K-562 (RRID:CVCL_0004) were obtained from ATCC. The metastatic neuroblastoma cell line BM derived from the IGR-N-91 model (RRID:CVCL_8883) was obtained in a collaborative work with Dr Jean Bénard, Gustav Roussy Institute, France [17]. The immortalised human alveolar epithelium cell line CI-hAELVi (RRID:CVCL_C1M0) was acquired from InSCREENex GmbH (Braunschweig, Germany). All cell lines were cultured at 37 °C in a 5% CO_2_ atmosphere, in the appropriate medium according to the provider’s instructions. For adherent cell lines, once the monolayer reached 80% confluence, cells were washed with sterile PBS and subsequently detached by treatment with 0.05% trypsin in PBS (Gibco, Grand Island, NY, USA). They were then washed with sterile PBS before being used for RNA extraction or immunofluorescence. For the suspension cell line (K562), the cells were centrifuged for 5 min at 1500 rpm and washed with sterile PBS prior to subsequent RNA extraction.

All lung cell lines were analysed by RT-PCR to assess *GALNT13* expression, while A549 and hAELVi cells were used in immunofluorescent experiments as controls to confirm *GALNT13* and cytokeratin staining, also confirming specificity of anti-GalNAc-T13 antibody.

### 4.3. RNA Extraction and cDNA Synthesis

Total RNA was extracted with TRIzol™ (Invitrogen, Thermo Fisher Scientific, South San Francisco, CA 94080, USA) as recommended by the manufacturer. One microgram of RNA was reverse transcribed in a 20 μL reaction containing 4 μL 5× buffer, 100 mM DTT, 200 U M-MLV reverse transcriptase, 2 μL each dNTP, and 200 ng random hexamers (SuperScript™ IV RT Invitrogen, Thermo Fisher Scientific, South San Francisco, CA, USA). The reaction was incubated at 25 °C for 10 min, 37 °C for 60 min, 85 °C for 5 min and stored at −20 °C.

cDNA quality was assessed by PCR amplification of a 600 bp β2-microglobulin fragment using primers B2M-S (5′-ATGTCTCGCTCCGTGGCCTTAG-3′) and B2M-AS (5′-AAGTTGCCAGCCCTCCTAGAGC-3′). PCR was performed in 10 mM Tris-HCl (pH 8.8), 50 mM KCl, 2 mM MgCl_2_, 200 µM each dNTP, 300 nM each primer, and 1 U Taq DNA polymerase recombinant (Invitrogen™, Thermo Fisher Scientific, South San Francisco, CA, USA). Cycling conditions were 95 °C for 5 min, 35 cycles of 95 °C, 62 °C, and 72 °C for 1 min each, and a final extension at 72 °C for 5 min.

### 4.4. Nested RT-PCR (nRT-PCR) and Digital RT-PCR (dRT-PCR) for GALNT13 Expression Assessment

First-strand cDNA synthesised from total RNA was used as the template for the primary PCR with outer *GALNT13* primers (GALNT13-F, 5′-ACATCTATCCGGACTCCC-3′; T13_XhoI_Rev, 5′-TCATGTGCCCAAGGTCATGTTCC-3′). The 25 μL reaction contained 10 mM Tris-HCl (pH 8.8), 50 mM KCl, 3 mM MgCl_2_, 200 μM each dNTP, 300 nM each primer, and 1 U Taq DNA polymerase recombinant (Invitrogen™, Thermo Fisher Scientific, South San Francisco, CA, USA). Cycling conditions were 94 °C for 5 min; 35 cycles of 94 °C for 30 s, 60 °C for 30 s, and 72 °C for 1 min; and a final extension at 72 °C for 5 min. One microliter of the first-round product was used for nested PCR under similar conditions, except with 2 mM MgCl_2_, an annealing temperature of 56 °C and inner *GALNT13* primers (T13-10F, 5′-AAATCCGAACCGATGACTTG-3′; T13-11R, 5′-TAGGCACCATTTTGTCTTCTT-3′). The nested PCR amplified a specific internal fragment to enhance detection sensitivity and specificity. Products were resolved on 2.5% agarose gels stained with ethidium bromide and visualised under UV light.

*GALNT13* expression in cell lines and clinical samples was quantified by digital RT-PCR (QIAcuity Digital PCR System, Qiagen^®^). Each 40 μL reaction contained 2 μL cDNA, 13.3 μL 3× EvaGreen PCR Master Mix (QIAcuity^®^ EG PCR Kit, Qiagen, Hilden, Germany), and 200 nM each primer (GALNT13-F, 5′-ACATCTATCCGGACTCCC-3′; GALNT13-R, 5′-GGCCCATGTTGTCTAAA-3′). Cycling conditions were 95 °C for 2 min, followed by 35 cycles of 94 °C for 15 s, 56 °C for 15 s and 72 °C for 5 s, and a final step at 40 °C for 5 min. Data were analysed using the QIAcuity Software Suite^®^ version 3.2.

### 4.5. CTC Enrichment Using the IsoFlux^TM^ System

CTCs were enriched using the IsoFlux™ Circulating Tumour Cell Enrichment Kit and IsoFlux System (Fluxion Biosciences, Alameda, CA, USA) following the manufacturer’s instructions. Briefly, 5 mL of peripheral blood was diluted 1:10 in 1× Red Blood Cell Lysis Solution (Miltenyi Biotech™, Bergisch Gladbach, Germany), incubated for 10 min at room temperature, centrifuged at 300× *g* for 10 min, and washed with 5 mL PBS. The pellet was resuspended in 500 μL binding buffer (BB), transferred to a Protein LoBind™ tube (Eppendorf, Hamburg, Germany), and incubated for 5 min with 40 μL FC Blocker. Then, 40 μL magnetic beads conjugated to anti-EpCAM antibody were added, and samples were incubated for 2 h at 4 °C with gentle agitation. After incubation, samples were processed on the IsoFlux™ System for automated CTC enrichment. Captured cells were recovered in BB, fixed with 3.7% paraformaldehyde:BB (1:1) for 20 min at room temperature, and stored in BB at 4 °C until use.

### 4.6. Characterisation of CTCs by Confocal Microscopy

Immunobeads containing captured cells were incubated twice with papain (LK003176, Worthington Biochemical, Lakewood, NJ, USA) for 20 min at 37 °C under agitation (650 rpm), then washed with PBS containing 1% BSA. The supernatant, collected in a Protein LoBind™ tube using a magnet, was centrifuged, and the recovered CTCs were permeabilised with 0.2% Triton X-100 for 5 min at room temperature, followed by PBS–BSA washes. GalNAc-T13 was detected using the monoclonal antibody T13.5 [19] and a goat anti-mouse IgG H&L Alexa Fluor^®^ 555–conjugated secondary antibody (Abcam, ab150114, Cambridge, UK). Cytokeratin (CK) was stained with FITC-conjugated anti-pancytokeratin monoclonal antibody (clone CK3-6H5, 130-060-301, Miltenyi Biotec, Bergisch Gladbach, Germany), and nuclei were counterstained with Hoechst 33342 (Miltenyi Biotech, 130-111-569). After PBS–BSA washing, cells were cytocentrifuged at 1000 rpm for 10 min, air-dried, and examined by confocal microscopy using a Zeiss LSM 710 laser scanning confocal microscope (Carl Zeiss Microscopy GmbH, Jena, Germany) at the Microscopy Unit of the GENYO (Granada, Spain).

### 4.7. Statistical Analysis

The association between *GALNT13* expression and clinicopathological variables in lung cancer patients and controls was analysed using the chi-square test or Fisher’s exact test. A *p* < 0.05 was considered statistically significant. Survival curves were estimated by the Kaplan–Meier method and compared using the Mantel–Cox log-rank test. The proportional hazards assumption was evaluated using Schoenfeld residuals and −log(−log S(t)) plots. Longitudinal changes in *GALNT13* expression were analysed using generalised estimating equations (GEE) to account for multiple paired comparisons contributed by the same patient. For each pair, the change in *GALNT13* expression was expressed as the natural logarithm of the ratio of the follow-up value to the initial sample value. Patient identifier was specified as the clustering variable, with an exchangeable within-patient correlation structure and robust standard errors. Estimates were obtained separately for each clinical status at follow-up (objective response, stable disease, or progressive disease), then back-transformed and reported as geometric follow-up-to-initial ratios with 95% confidence intervals. All tests were two-sided, with *p* < 0.05 considered statistically significant. To assess whether *GALNT13* positivity was associated with overall survival after adjustment for relevant clinical factors, multivariable Cox regression models were fitted separately for *GALNT13* status determined by nRT-PCR and dRT-PCR. Each model included *GALNT13* status, disease stage (I–IV), age as a continuous variable, sex, and smoking status. Hazard ratios (HRs) and 95% confidence intervals (CIs) were reported. The proportional hazards assumption was assessed using Schoenfeld residuals.

## Figures and Tables

**Table 1:** *GALNT13* expression in lung cancer lines.

Cell Line	Histological Type	*GALNT13* Expression
A549	ADC (NSCLC)	Positive
NCI-H1755		Positive
NCI-H1650		Positive
NCI-H838		Negative
NCI-H1975		Negative
SK-MES-1	SCC (NSCLC)	Negative
NCI-H1703		Positive
NCI-H526	SCLC	Positive
H69AR		Positive
NL-20	Immortalized bronchial cells	Weak

ADC, adenocarcinoma; NSCLC, non-small cell lung cancer; SCC, squamous cell carcinoma; SCLC, small cell lung cancer.

**Table 2:** Sensitivity analysis of *GALNT13* detection by nested RT-PCR and digital RT-PCR using serial dilutions of *GALNT13*-expressing A549 cells in *GALNT13* non-expressing K562 cells.

Number	Number of A549 Cells	Number of K562 Cells	Ratio	nRT-PCR	dRT-PCR
1	30,000	3 × 10^6^	1/100	+	+
2	15,000	3 × 10^6^	1/200	+	+
3	7500	3 × 10^6^	1/400	+	+
4	3750	3 × 10^6^	1/800	+	+
5	1875	3 × 10^6^	1/1600	+	+
6	938	3 × 10^6^	1/3200	+	+
7	469	3 × 10^6^	1/6400	+	±
8	234	3 × 10^6^	1/12,800	±	−
9	117	3 × 10^6^	1/25,600	−	−
10	58	3 × 10^6^	1/51,200	−	−
11	29	3 × 10^6^	1/102,400	−	−
12	14	3 × 10^6^	1/204,800	−	−

A549, lung adenocarcinoma cell line; K562, chronic myelogenous leukaemia cell line; nRT-PCR, nested RT-PCR; dRT-PCR, digital RT-PCR; + positive; − negative; ± weak signal.

**Table 3:** Association between *GALNT13* expression and clinicopathological variables in NSCLC patients.

Variable	n (%)	nRT-PCR (+)	nRT-PCR (−)	dRT-PCR (+)	dRT-PCR (−)
Patients	143	86 (60)	57 (40)	76 (53)	67 (47)
Age	Median 65 Range 32–84				
Sex	F: n = 49 (34)	28 (57)	21 (43)	22 (45)	27 (55)
	M: n = 94 (66)	58 (62)	36 (38)	54 (57)	40 (43)
Histology	ADC (n = 76)	45 (59)	31 (41)	35 (46)	41 (54)
	SCC (n = 39)	26 (67)	13 (33)	26 (67)	13 (33)
	Others (n = 28)	15 (54)	13 (46)	15 (54)	13 (46)
T	T1 (n = 10)	3 (30)	7 (70)	2 (20)	8 (80)
	T2 (n = 41)	20 (49)	21 (51)	20 (49)	21 (51)
	T3 (n = 59)	42 (71)	17 (29)	38 (64)	21 (36)
	T4 (n = 33)	21 (64)	12 (36)	16 (48)	17 (52)
N	N0 (n = 33)	20 (61)	13 (39)	16 (48)	17 (52)
	N+ (n = 110)	66 (60)	44 (40)	60 (55)	50 (45)
M	M0 (n = 64)	37 (58)	27 (42)	30 (47)	34 (53)
	M+ (n = 79)	49 (62)	30 (38)	46 (58)	33 (42)
Stage	I–IIIA (n = 36)	18 (50)	18 (50)	13 (36)	23 (64)
	IIIB–IV (n = 107)	68 (64)	39 (36)	63 (59)	44 (41)

NSCLC, non-small cell lung cancer; ADC, adenocarcinoma; SCC, squamous cell carcinoma; nRT-PCR, nested RT-PCR; dRT-PCR, digital RT-PCR.

## Data Availability

The original contributions presented in this study are included in the article. Further inquiries can be directed to the corresponding author.
